# Circular RNAs in metabolic health: bridging the gap between molecular biology and therapy

**DOI:** 10.1038/s41419-026-08450-5

**Published:** 2026-02-25

**Authors:** Yutong Huang, Tianle He, Jundan Zheng, Jiaxin Chen, Zhenguo Yang

**Affiliations:** Laboratory for Bio-feed and Molecular Nutrition, College of Animal Science and Technology, Southwest University, Chongqing, 400715 China

**Keywords:** Obesity, RNA

## Abstract

Recent advances in obesity research have shifted focus toward biological mechanisms, paralleling progress in pharmacotherapy. Fat browning—the conversion of white to brown adipocytes—emerges as a promising therapeutic strategy. Circular RNAs (circRNAs), stable non-coding RNAs with regulatory functions, are now recognized as key modulators of this process through organelle-mediated mechanisms. This review synthesizes current understanding of circRNA biogenesis and their roles in fat browning, particularly their interactions with mitochondria and endoplasmic reticulum in lipid metabolism. We highlight their capacity to encode peptides and regulate metabolic pathways, positioning circRNAs as potential precision therapeutics. While preclinical studies demonstrate mechanistic efficacy, clinical translation requires addressing delivery challenges and tissue-specific effects. This biological perspective advances obesity treatment paradigms beyond simplistic energy-balance models, mirroring the evolution seen in pharmacotherapeutic development.

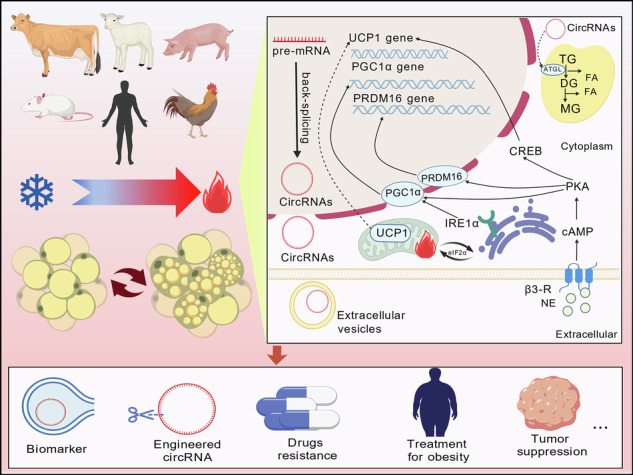

## FACTS

- Circular RNAs (circRNAs) interact with mitochondria and endoplasmic reticulum, affecting adipose browning and metabolic reprogramming.
- CircRNA regulatory networks aid in understanding lipid homeostasis-associated metabolic pathways.
- CircRNA interactions with key metabolic pathways are potential targets for precise treatment of obesity and metabolic diseases.
- Low delivery efficiency and poor tissue specificity of circRNAs restrict their use in regulating lipid metabolism-related diseases.

## Open questions

- What are the precise molecular mechanisms by which specific Circular RNAs (circRNAs) regulate mitochondrial function and ER stress during fat browning?
- How do circRNA-encoded peptides functionally contribute to lipid metabolism and energy homeostasis in vivo?
- Do circRNAs exhibit distinct regulatory patterns across white/brown/beige adipocytes, and how to leverage this for therapy?
- Can circRNA-based therapeutics be efficiently delivered to adipose tissue in a safe and tissue-specific manner?

## Introduction

**Fig. 1:**
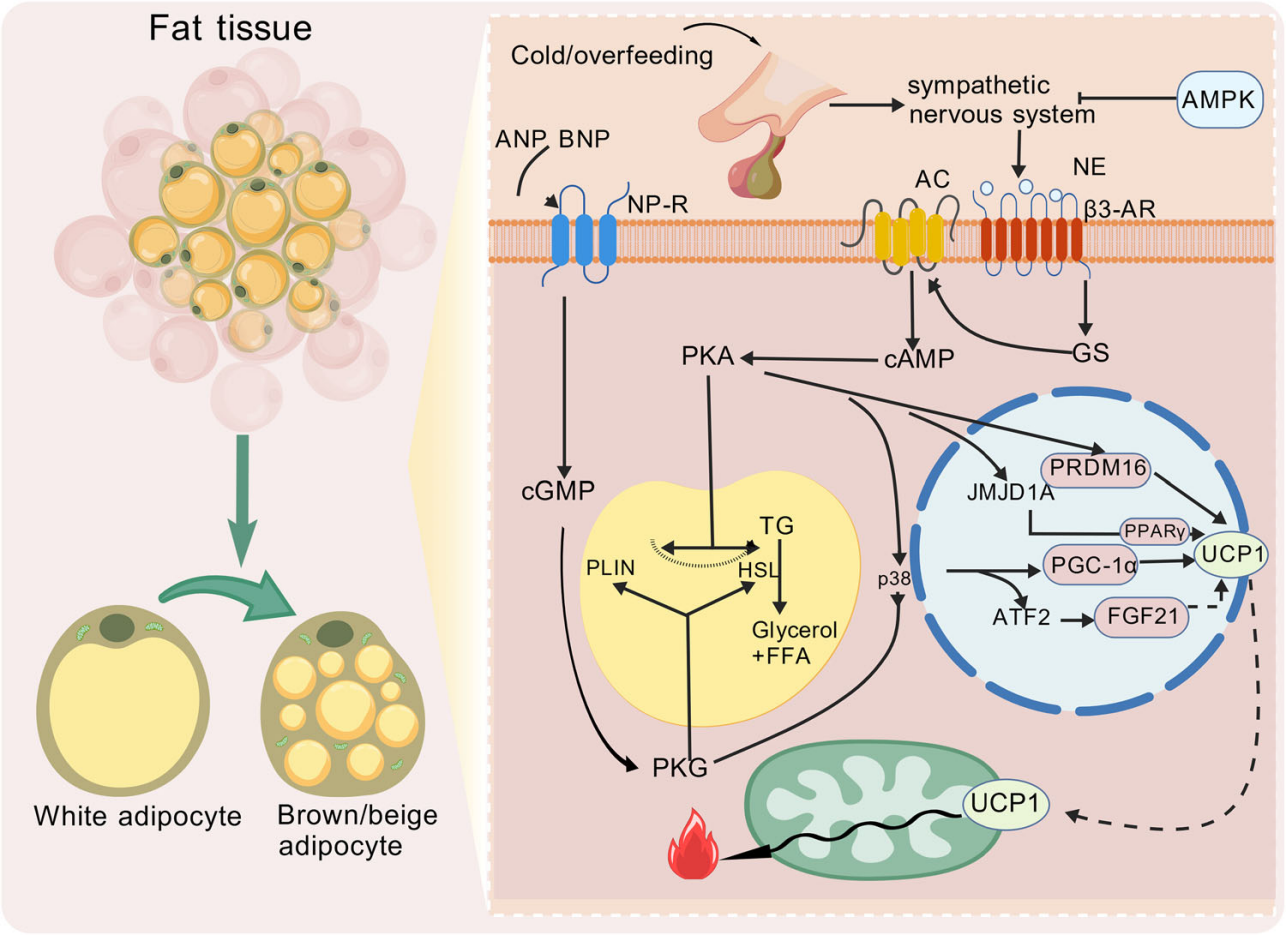
Adipocyte type transformation and its potential regulatory mechanisms. Cellular changes and associated mechanisms of fat transformation: fat browning is regulated by the central nervous system, peripheral nervous system, transcription factors, environmental factors, pharmacological agents. Created with BioGDP.com.

Within the mammalian energy metabolism, adipose tissue exhibits distinctive biological significance due to its anatomical distribution and functional specialization. White adipose tissue (WAT), predominantly localized in subcutaneous and visceral depots [1], dynamically stores and releases energy. A large lipid droplet with a single cavity and low mitochondrial density makes it a pivotal effector in obesity-associated metabolic diseases [2, 3]. By contrast, brown adipose tissue (BAT), primarily situated in interscapular brown adipose tissue and deep cervical regions [4], has multiple lipid droplets and high mitochondrial density rich in uncoupling protein 1 (UCP1). It increases energy expenditure and maintains body temperature by enhancing non-shivering thermogenesis, resulting in decreasing weight gain [5–7]. Emerging evidence highlights a unique adipocyte subtype-beige adipocytes [8], which are ontogenetically related to white adipocytes and located within WAT [4]. Under certain physiological stimuli, WAT undergoes phenotypic remodeling to generate beige adipocytes-a metabolically adaptive phenomenon termed fat browning [9, 10], which is triggered by cold stimuli, overfeeding, or exercise (irisin secretion) [5]. Moreover, it is regulated by many factors, including the central nervous system, peripheral nervous system, key factors of transcription, environment, and medicine. Recently, researchers proposed that GABAergic neurons in the dorsal-medial nucleus of the hypothalamus can reduce the activity of Protein Kinase A (PKA) subunits and increase sympathetic nerve activity, resulting in fat browning and the expression of thermogenic genes [11, 12]. In addition, after the stimulation of the β-adrenergic receptor (β3-AR) on adipocytes, the downstream signaling pathways are activated, including the cAMP-PKA-CREB, β-AR-mTOR-Lipin1 axis and AMPK/SIRT1 pathway, which enhances fatty acid oxidation and mitochondrial function [7, 13]. After the stimulation of AMPK, It not only modulates the activity of PGC1α and enhances the expression of UCP1, but also facilitates the demethylation of the PR domain-containing 16 (PRDM16) promoter, thereby directly regulating fat browning [14, 15]. These processes are shown in Fig. 1. However, current research also points out that a novel UCP1-independent thermogenic mechanism-a process distinct from classical UCP1-mediated thermogenesis through ATP-dependent futile cycling [16], which is associated with a P2 adipocyte subpopulation within brown/beige adipose depots.

According to current research, the disorder of fat metabolism is the core pathophysiological mechanism of many metabolic diseases, including obesity and its complications. Obesity is a global health issue with a sharp increase in the case rate, which will reach 25% by 2025 predicted by the World Health Organization (WHO) [17]. It is estimated that ~1.5 billion adults in the world are overweight, including 200 million men and 300 million women [18]. For obese organisms, hypertrophy, hyperplasia, inflammatory response[19] and abnormal secretion of adipokines can occur[20], which may contribute to systemic metabolic disorders. Besides, obesity is related to type 2 diabetes, hypertension, hyperlipidemia, chronic kidney disease, cardiovascular diseases, obstructive sleep apnea, and many types of cancer [21], especially cardiovascular disease and the occurrence and development of non-alcoholic fatty liver disease (NAFLD) which may do serious harm on organisms[18, 22, 23]. By inducing the conversion of fat into brown adipocytes, the symptoms of these diseases can be relieved, which maintains the stability of fat metabolism [11, 24].

However, with the deepening of the research on fat browning, more and more new perspectives have emerged. In the field of molecular biology, circular RNAs (circRNAs) have become a hot topic in the field of metabolism because of their special structure and function. In fat metabolism, research about circRNAs has gradually revealed its key role in adipose tissue development, adipocyte differentiation, and fat browning. In addition, the abnormal expression of circRNAs in obesity and its related metabolic diseases further shows their potential as biomarkers and therapeutic targets. In this review, we pay attention to exploring the role of circRNAs in promoting the browning of WAT, aiming to build a molecular foundation for the treatment of obesity-related diseases, propose novel treatment strategies based on circRNAs, and deeply discuss the technical means that can be applied in the future.

## Overview of circRNAs

**Fig. 2:**
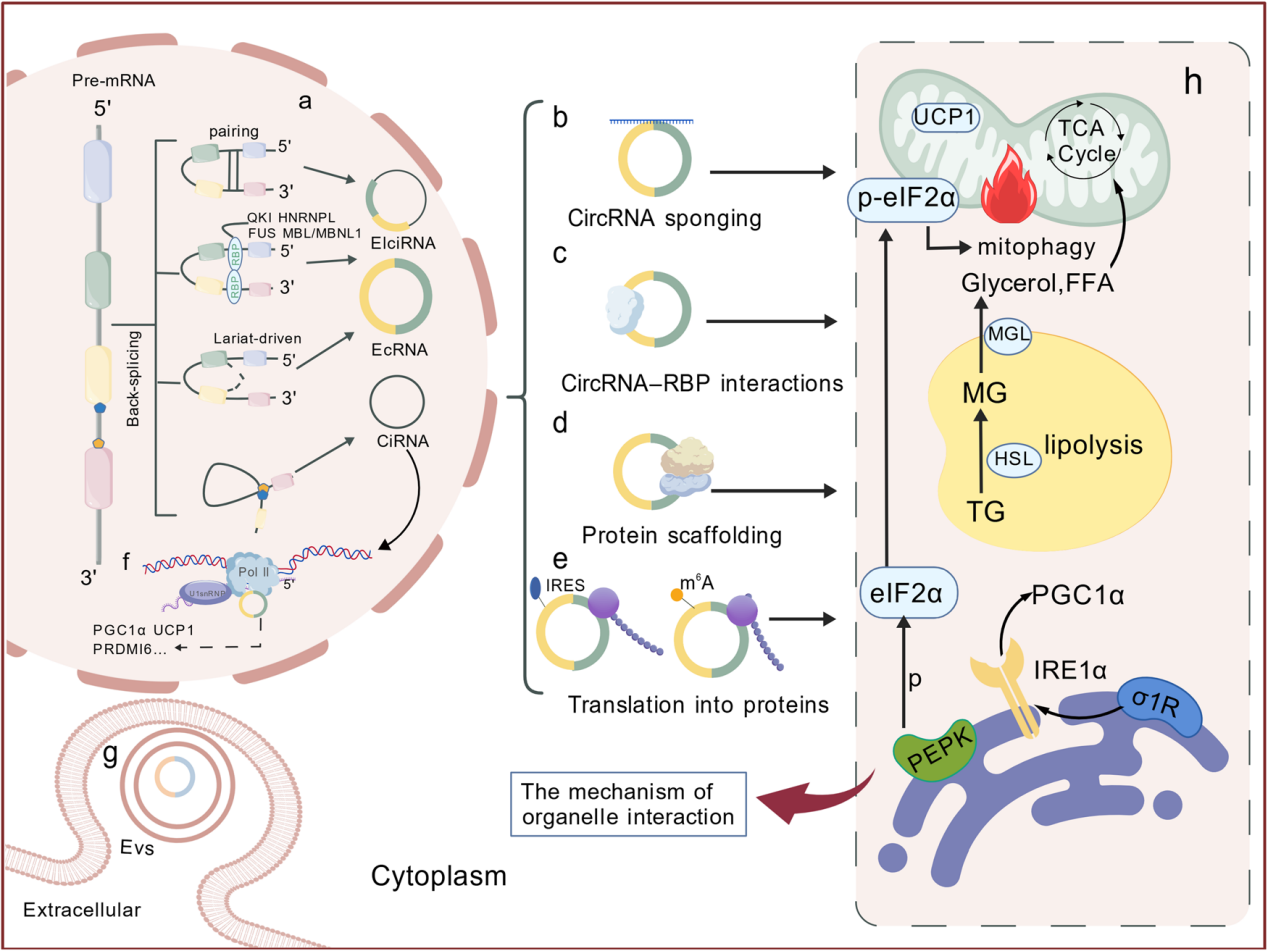
Circular RNAs (CircRNAs) play an important role in cell life. **a** CircRNAs are formed in the nucleus as shown in figure, **b** and the ways in which they function in the cell include: Spongiosis of circRNAs, **c** interaction of circRNAs with proteins, **d** role of circRNAs as protein scaffoldings, **e** potential translational functions of circRNAs, **f** regulation of transcription processes in the nucleus by circRNAs, and so on. **g** CircRNAs are secreted extracellular by way of exosomes. **h** circRNAs regulate the interaction of organelles related to fat browning. Created with BioGDP.com.

As a representative of non-coding RNAs, circRNAs remodel the biological significance of RNA through their unique covalently closed annular structure [25], which also challenges the central dogma. Since the initial identification of endogenous circRNAs in viroids by Sanger’s group in 1976 [26], these covalently closed RNA molecules have captured scientific attention. Subsequent advancement in RNA sequencing (RNA-seq) technology revealed their widespread distribution across eukaryotes, exhibiting tissue-specific expression [27]. CircRNAs are produced by a back-splicing of precursor mRNAs [28–31], forming a covalently closed topology with 3’-5’ phosphodiester bonds to confer intrinsic nuclease resistance [27, 32–35]. The structural stability and topological compactness of circRNAs enable their selective incorporation into exosomes, which conduct a long-distance intercellular communication [36]. Furthermore, the architecture of circRNAs gives them a distinct molecular interaction capability compared to linear RNAs, establishing a structural foundation for their regulatory ability. At the post-transcriptional stage, certain circRNAs exhibit miRNA-binding activity through sequence complementarity [37–39]. An example is circular RNA sponge for miR-7 (ciRS-7), which demonstrates potent miRNA sequestration via 70 evolutionarily conserved binding sites [33]. It is its circular structure that shows a spatial possibility for other molecules to bind. Besides, circRNAs can absorb specific proteins (such as RNA binding protein) to work, including enhancing and disarming the interaction between proteins, isolating specific proteins, and forming a circRNA-protein-nucleic acid ternary complex to affect gene expression [40]. In hepatocellular carcinoma, circBACH1 mechanistically interacts with Human antigen R (HuR) protein to alter its subcellular distribution and modulate p27 expression [41]. In addition, circ-Foxo3 orchestrates cell cycle arrest by serving as a structural scaffold through circular conformation, which facilitates the simultaneous recruitment of cyclin-dependent kinases 2 (CDK2) and cyclin-dependent kinase inhibitor 1 A (p21) to assemble a ternary complex [42]. This structure-function coupling principle extends to metabolic regulation-circACC1 and exemplifies precision control by simultaneously anchoring AMPK β/γ subunits, thereby conferring allosteric enhancement of holoenzyme stability and catalytic efficacy [43]. Emerging paradigm-shifting studies have redefined the RNA regulatory landscape, unveiling circRNAs non-canonical role within the central dogma [44]. While classical interpretations emphasize the effect of coding RNAs in genetic information flow, contemporary findings suggest circRNAs as transcriptional modulators-exemplified by direct interactions with U1 snRNP/RNA polymerase II complexes to enact chromatin-level transcriptional regulation [44, 45]. At the translational level, circRNAs circumvent the linear constraints of mRNAs through structural innovation, conducting translating via internal ribosome entry sites (IRES) or m6A modifications [46–54]. This mechanistic plasticity is proved by circ-FBXW7-derived 185-amino acid polypeptide that suppresses the development of glioma [55]. The biogenesis and multifunctional landscape of circRNAs are schematized in Fig. 2.

## The role of circRNAs in fat browning

### CircRNAs act as a sponge for miRNAs to regulate fat browning

**Fig. 3:**
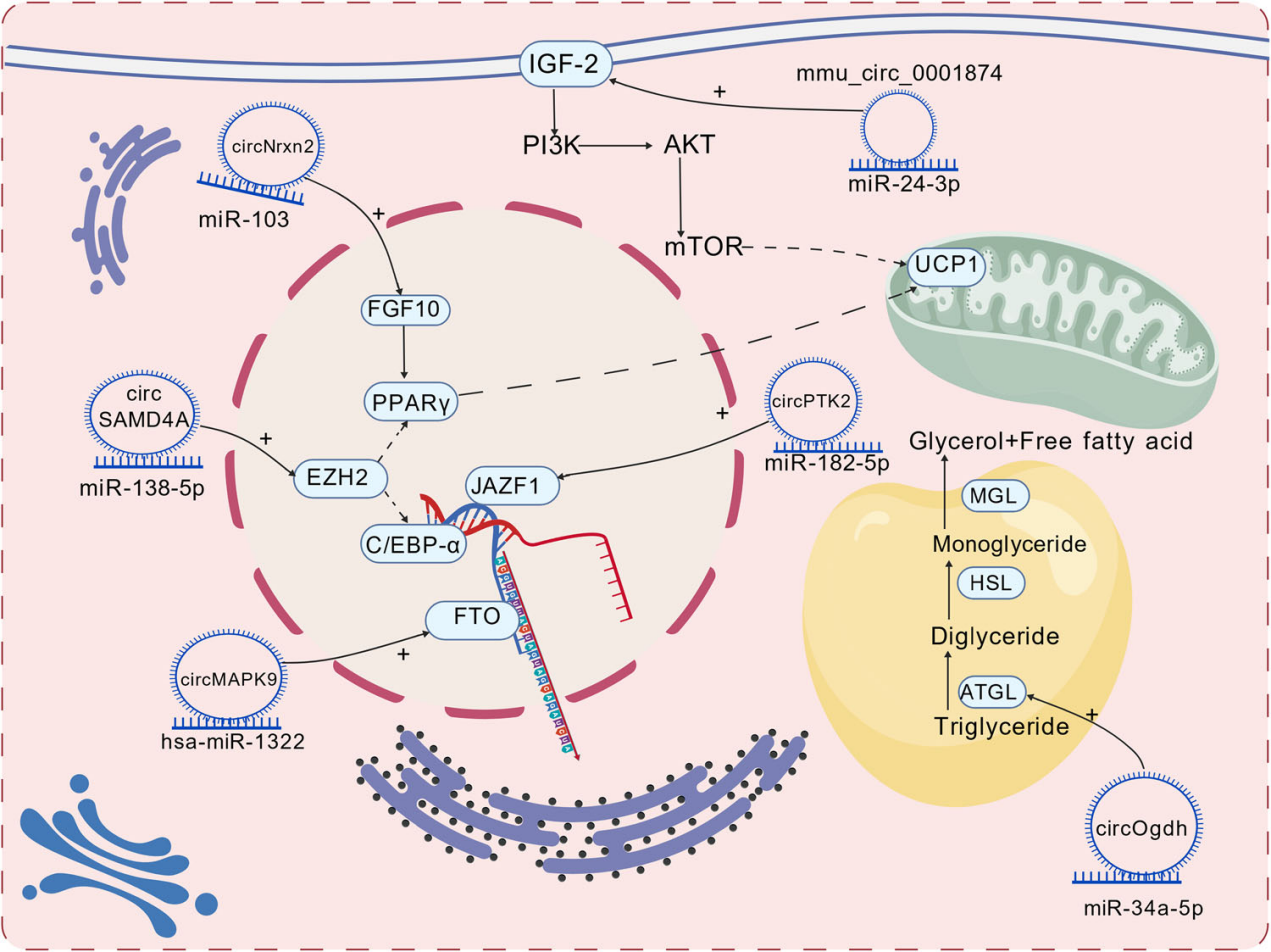
Mechanistic regulation of fat browning by circRNAs via miRNA sponging in mammalian cells. The processes that different circRNAs are involved in regulating the fat browning process (in human or mouse cells) through spongiosis. Created with BioGDP.com.

In recent years, most circRNAs contain response elements (MREs) of responding miRNAs, acting as ceRNAs by serving as a sponge for microRNAs (miRNAs), which influences the expression of target genes[33, 56]. This mechanism allows circRNAs to regulate the process of fat browning. Researchers identified 165 upregulated and 79 downregulated circRNAs in BAT of C57BL/6 J mice through RNA sequencing (RNA-seq)[57]. Among these, a novel circRNA called circOgdh was highly expressed in BAT, which is capable of adsorbing miR-34a-5p and then upregulating the expression of Atgl, the key lipolysis gene and plays an important role in enhancing the lipolysis of brown adipocytes[57]. In recent years, differentially expressed circRNAs of host genes have been discovered to gather in signal pathways related to fat browning, including the AMPK, HIF-1, AKT, and mTOR signaling pathways through differential expression analysis of GO and KEGG enrichment. Specific circRNAs regulate signal pathways related to glucose and lipid metabolism, which drives lipid metabolism reprogramming and enhances heat production. Notably, the AKT signaling pathway lies at the junction of liver immunity and cell metabolism. Persistent activation of AKT/mTOR leads to increased fat production, oxidative stress, and hepatocyte proliferation, which is conducive to creating the microenvironment for liver cancer[58]. Additionally, studies have shown that the AKT signaling pathway can also affect CRYAB by promoting M2 polarization through Akt1/mTOR, and long-term polarization inhibition leads to the dysfunction of CD8 + T cells[58, 59]. Furthermore, if NF-κB is not inhibited at this point, the formation of the tumor microenvironment will be rapidly driven[58]. Moreover, in recent years, researchers have discovered that SCARF1 promotes Kupffer cell M2 polarization through the calcium-dependent PI3K-AKT-STAT3 signaling pathway, and it also plays an important role in shaping the microenvironment of liver cells[60]. Accordingly, unraveling the cell-specific roles of the AKT pathway across diverse hepatic cell subsets and its regulatory interplay with circRNAs (as miRNA sponges) is imperative for advancing targeted therapeutic approaches. A promising strategy could involve engineering a circRNAs-miRNAs-AKT axis within hepatic cells or organoids, which would potentiate systemic metabolic regulation while mitigating the risk of inducing tumorigenic or metabolic adverse effects in hepatocytes. A research from Shao et al.[61] revealed that mmu_circ_0001874 in vivo reduces fat accumulation in obese mice and regulates lipid metabolism through the miR-24-3p/Igf2/PI3K-AKT-mTOR axes, offering potential targets for human obesity treatment. Another notable study indicated that CircRNF111 in bovine adipocytes increases peroxisome proliferator-activated receptorγ (PPARγ) expression by adsorbing miR-27a-3p, thereby promoting adipocyte differentiation and ultimately upregulating the expression of the UCP1 gene[62]. Additionally, according toa current study on the PPARγ gene, it is revealed that circPPARγ plays a crucial regulatory role in bovine fat, where it binds with miR-92a-3p to promote adipocyte differentiation[63]. Furthermore, based on the research on bovine fat metabolism, it is found that circBDP1 acts as a sponge of miR-181b/miR-204 to regulate their expression. As miR-181b and miR-204 target SIRT1 and TRARG1—genes related to adipocyte thermogenic capacity-circBDP1 indirectly influences lipid metabolism[64]. Similarly, in chickens, circDOCK7 promotes the proliferation and differentiation of belly preadipocytes through the gga-miR-301b-3p/ACSL1 axis, which involves adipocyte maturation and metabolic capacity[65]. In studies on rats, differentially expressed circRNAs have been found to have shared miRNA binding sites through prediction of circRNAs-miRNAs interactions, among which circ-ATXN2 could influence adipose tissue function and aging by interacting with miRNAs[66]. According to new evidence, it is suggested that circRNAs could become a breakthrough for treating complex human metabolism-related diseases. In human obesity research, CircMAPK9 decreases the inhibitory effect on FTO and promotes adipogenesis by competitively binding to hsa-miR-1322, finally causing obesity. In addition, scientists discovered circSAMD4A could act as a sponge of miR-138-5p to increase EZH2 expression and promote adipocyte differentiation, thus affecting lipogenesis in obese patient[67]. In a study using high-fat diet (HFD) mice as experimental subjects, Zhang et al.[68] discovered that through the interaction with miR-103, circNrxn2 inhibits its activity and upregulates FGF10 expression, which is a key factor of WAT browning. Many studies have described how circRNAs in different species act as sponges to regulate the process of adipogenesis in the body (Table 1). Some specific circRNAs regulate the metabolic phenotype, which is similar to the Warburg effect, to promote glycolysis. In research by Zhong et al.[69] circ235 can release inhibition of MCT4 through adsorbing miR-330-5p, which promotes glycolysis and adipogenesis. The mechanism by which circRNAs regulate the process of fat browning through sponge-like effect is shown in Fig. 3. Through the current research on multispecies circRNAs, the status of cross-species research on metabolic reprogramming is gradually important. However, it is a pity that although there have been many studies on the sponge effect of circRNAs, there has been no research specifically focusing on the adaptability of circRNAs across species. Some circRNAs exist only in certain species, and the homologous circRNAs in other species cannot be guaranteed to have similar effects.

**Table 1:** List of circRNAs involved in fat Browning or fat metabolism processes through spongiosis.

CircRNAs	Corresponding miRNA	Species	Mode of action	Direction	References
circOgdh	miR-34a-5p	Mouse	Lipolysis	↑	[57]
circADAMTS16	miR-10167-3p	Cattle	Preadipocyte differentiation	↓	[184]
circBDP1	miR-181b/miR-204	Cattle	Fat browning	↑	[64]
circDOCK7	gga-miR-301b-3p/ACSL1	Chicken	Preadipocyte proliferation and adipodifferentiation	↑	[65]
circ-ATXN2	—	Rat	Lipogenesis	↑	[66]
circMAPK9	hsa-miR-1322	Human	Lipogenesis	↑	[185]
circSAMD4A	miR-138-5p	Human and mouse	Adipose precursor differentiation and lipogenesis	↑	[67]
circNrxn2	miR-103	Mouse	Lipobrowning	↑	[68]
mmu_circ_0001874	miR-24-3p	Mouse	Fat metabolism	↑	[61]
circRNF111	miR-27a-3p	Cattle	Fat metabolism	↑	[62]
circPPARγ	miR-92a-3p	Cattle	Fat metabolism	↑	[63]
circFLT1	miR-93	Cattle	Adipocyte proliferation and differentiation	↑	[186]
circPTK2	miR-182-5p	Human and mouse	Promotes lipolysis	↑	[187]
circITGB1	miR-23a	Sheep	Promote adipocyte proliferation and differentiation	↑	[188]
circBTBD7	miR-183	Cattle	Lipogenesis inhibition	↓	[189]
circMEF2C (2, 3)	miR-383/miR-671-3p	Pig	Lipogenesis inhibition	↓	[190]

### CircRNAs interact with proteins to modulate metabolism

**Fig. 4:**
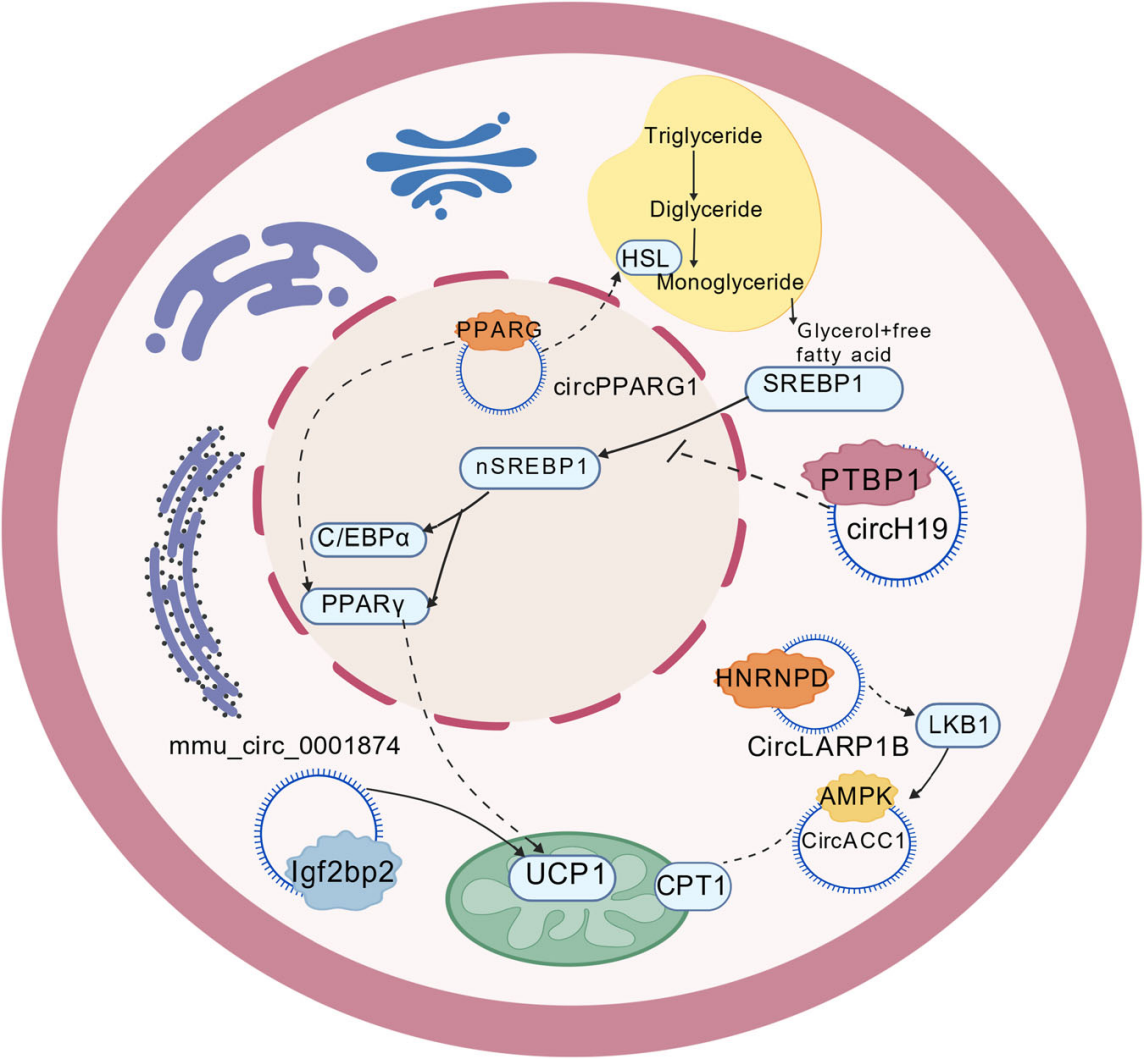
Specific circRNAs regulate fat metabolism and reprogramming by binding to metabolic enzymes or RNA binding protein (RBP). Created with BioGDP.com.

Recent studies have shown that some circRNAs can regulate biological activities by interacting with specific proteins related to glucolipid metabolism-related pathways, which drives lipid metabolism reprogramming (Table 2). RNA-binding proteins (RBPs) are the most common proteins during this process. It is suggested that the specific sequences of circRNAs can bind to RBPs, thereby influencing corresponding metabolic processes. In Zhu’s study, circRNA H19 in human adipose-derived stem cells (hADSCs) can regulate fat synthesis and differentiation by interacting with polypyrimidine tract binding protein 1 (PTBP1), which inhibits the migration of sterol regulatory element-binding protein 1 (SREBP1) from cytoplasm to nucleus[70]. Using intrahepatic cholangiocarcinoma (ICC) cell lines and related animal models as experimental subjects, relevant research found that circMBOAT2 interacts with PTBP1, which stabilizes the protein’s structure and promotes the output of FASN mRNA, playing a role in oxidative thermogenesis[71]. Additionally, Shao et al.[61] discovered that mmu_circ_0001874 in mouse cells can bind to insulin-like growth factor 2 (IGF-2), which upregulates the translation of UCP1 and enhances thermogenesis, thus promoting WAT browning. Ferroptosis, in terms of energy metabolism, can be associated with the process of fat metabolism and conversion. A study conducted by Xia on ferroptosis mechanisms showed that in mice with chronic obstructive pulmonary disease (COPD), N6-methyladenosine-modified circSAV1 recruits YTHDF1 to promote the translation of IREB2 mRNA, leading to the disorder of iron metabolism[72]. In the field of treatment of diseases, the interaction between circRNAs and RBP can be a breakthrough. Through inducing human Hepatocellular Carcinoma (HCC) models and hepatocellular carcinoma patient samples, scientists found that CircLARP1B influences the binding of HNRNPD protein to mRNA by recruiting HNRNPD, which indirectly activates the MPKA pathway and regulating lipid droplet formation[73]. CircRNA-RBP interaction also regulates metabolic phenotype, which is similar to the “Warburg” effect. Specific circRNAs can bind to proteins (e.g. circTICRR and HuR proteins) to regulate the stability of GLUD1, changing the pathway of glutamine decomposition, which regulates adipose differentiation[74]. Furthermore, specific circRNAs increase heat production by binding metabolic enzymes (e.g. AMPK and PGC-1α) that lead to fat metabolic reprogramming[43, 75]. In research by Li et al.[43] it is found that circACC1 can directly bind to the β1 and γ1 regulatory subunits of AMPK, promoting β oxidation and glycolysis. Several exonic circRNAs produced by PPARG can interact with the PPARG protein and inhibit the transcription of hormone-sensitive lipase (HSL), a protein involved in lipolysis[76, 77]. These mechanisms of action are shown in Fig. 4. Moreover, there are also some studies about the relationship between circRNAs and cell death inducing DFFA like effector a (CIDEA) family proteins, an important substance to regulate lipid droplet storage[78]. The knockdown of circArhgap5-1 in fat tissue results in a downregulation of CIDEA and suppression of lipid accumulation[79]. In addition to the above effects, circRNAs can also participate in the transcription of genes related to fat browning through interactions with key proteins. Current studies have shown that Exon-Intron CirRNAs (EIciRNAs) can influence the interaction between U1 snRNP and RNA Pol II, thereby affecting the generation of mRNA in metabolism[29]. Another study indicates that circCAPRIN1 in colorectal cancer (CRC) patient cells can activate the transcription of ACC1 by binding to STAT2, which influences tumor progression and lipid metabolism[80].

**Table 2:** List of circRNAs involved in fat browning or fat metabolism through interaction with proteins.

CircRNAs	Proteins acted	Species/Tissue	Mode of action	Direction	References
circRNAH19	PTBP1	Human adipose-derived stem cells	Adipogenesis and differentiation	↓	[70]
circMBOAT2	PTBP1	Intrahepatic cholangiocarcinoma (ICC) cells	Thermogenesis of brown fat cells	↑	[71]
circ_0001874	IGF-2	Mouse	Promotes fat browning	↑	[61]
circSAV1	YTHDF1	Mouse	Iron metabolism and energy metabolism	—	[72]
circLARP1B	HNRNPD	Human Hepatocellular Carcinoma (HCC) model	Formation of lipid droplets	↑	[73]
circArhgap5-2	—	Mouse	Adipogenesis	↑	[79]
EIciRNAs	U1 snRNP and RNA Pol II	—	Regulate transcription	—	[29]
circCAPRIN1	STAT2	Colorectal cancer (CRC) patient cells	Lipid metabolism	↑	[80]
circRNAs associated with PPARG	PPARG	—	Lipolysis	↑	[77]

CircRNAs are involved in epigenetic regulation to affect fat metabolism through binding to related enzymes. According to current evidence, circRNAs recruit proteins such as DNA methylases, histone modifying enzymes, etc. to chromatin, which may change the chromatin state of some genes associated with fat browning[40]. CircRNAs (e.g. **FECR1**) recruit a dioxygenase (TET1), which is key in the demethylation process[81]. Some circRNAs and proteins (e.g. STAT3) co-locate in DNA Methyltransferase (DNMT) promoters’ regions, interacting to regulate the epigenetics[81, 82]. Besides, circMRPS35 recruits lysine acetyltransferase 7 (KAT7) to the FOXO1/3a promoter area, which contributes to the acetylation of H4K5[83]. These epigenetic mechanisms will form a long-term memory of metabolic stress, which is important for organisms to live[84].

However, the interaction between circRNAs and proteins is bidirectional. In addition to the mentioned effects, proteins also conduct their action on the synthesis and degradation of circRNAs[76]. From the perspective of circRNAs biogenesis, RBPs (e.g. MBL and QKI) bind to the intron sequence on both sides of the extrons during circRNAs formation to form a dynamic regulatory complex[85], which enhances structural stability and splicing efficiency of genes related to fat browning, finally influence thermogenic metabolic pathway[29, 86]. According to the findings of Jia et al.[87] there are multiple domains in Drosophila GW182 and its human homologs (TNRC6A, TNRC6B, which TNRC6C) bind to circRNAs and broadly influence their stability, providing data support about regulation of circRNAs degradation. Dong et al.[88]. recently found that RBM3 binds to SCD-circRNA2, and the increasing expression of RBM3 in HCC is positively correlated with the expression level of SCD-circRNA2. In conclusion, the interaction between circRNAs and proteins is bidirectional and complex. Currently, there is no clear classification for this interaction. Moreover, the action sites of some circRNAs are still unknown. Future research should focus on a more precise level, by discovering their specific action sites to provide a basis for subsequent artificial intervention.

### CircRNAs encode proteins to regulate metabolic activities

Recent studies found that some circRNAs can be directly translated into small peptides, playing functional roles in cells[89, 90]. According to research by Jiang et al.[91] in gastric cancer tissues, circMAPK1 encodes a novel protein, MAPK1-109aa, which inhibits the activation of the MAPK signaling pathway. When it comes to fat metabolism, Wang et al.[92] suggested that in patients who have Non-Alcoholic Fatty Liver Disease (NAFLD), circ-SLC9A6 encodes a novel 126-amino acid peptide, SLC9A6-126aa, which is associated with lipid metabolism disorders. Consequently, gene knockdown of these circRNAs may provide a therapeutic way to treat the disease. Additionally, another notable study found that circ-CUX1 enhances fat metabolism reprogramming and mitochondrial function through encoding a 113-amino acid protein[93]. Due to the translation function and inherent stability of circRNAs, they can also be applied as vaccines. In the research of Qu et al.[94] circRNAs which have the ability to translate the SARS-CoV-2 receptor can be used as stable mRNA vaccines for disease prevention and control. Recent studies have also demonstrated that with specific engineering techniques, the in vitro translation of circRNAs can be long-lasting, which is currently being put into practice[95]. Therefore, future research may focus on applying this technique in various fields, such as enhancing the coding function of circRNAs in fat metabolism.

### Effects of circRNA cyclization mechanism on metabolic remodeling of circRNA of adipocytes

**Fig. 5:**
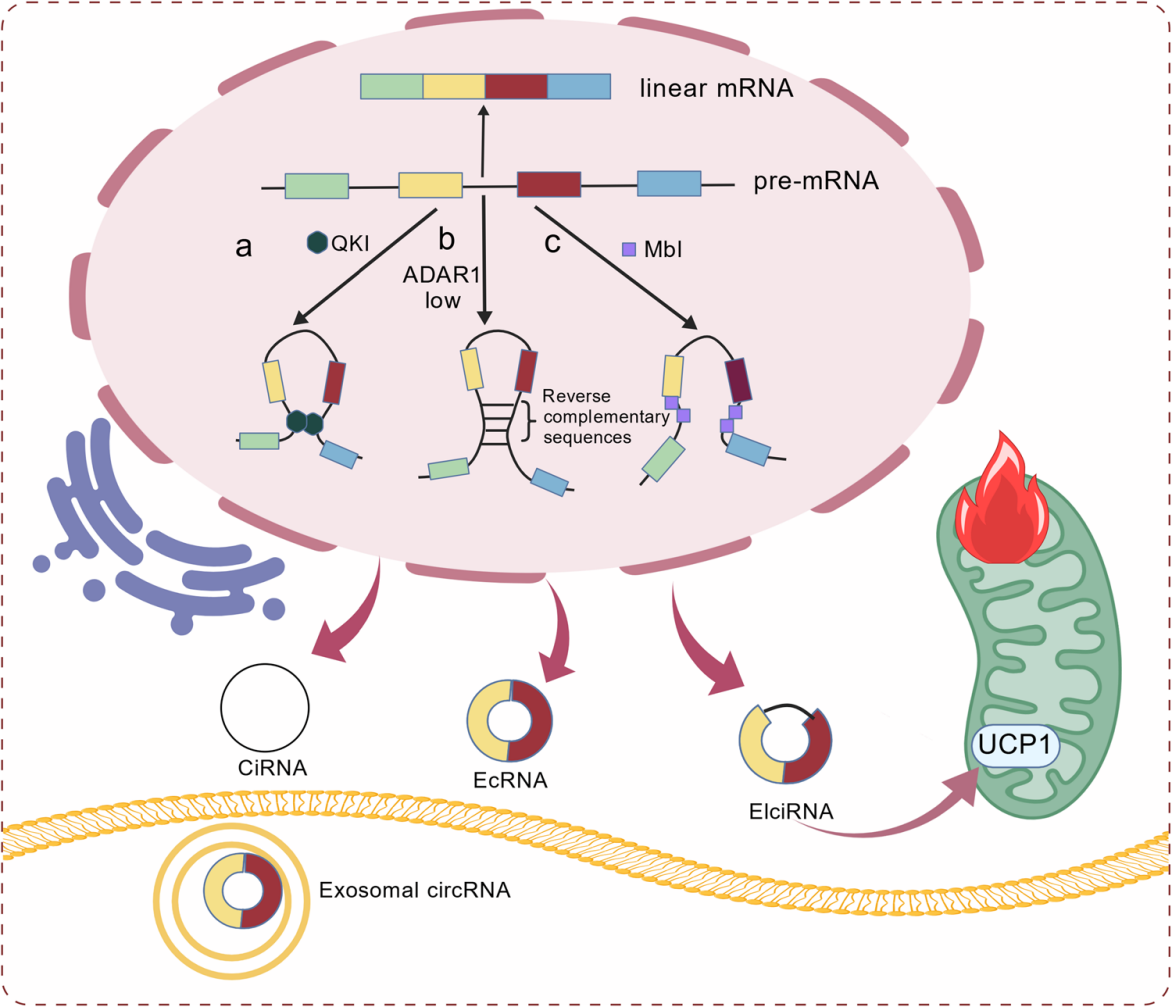
The loop formation of circRNAs enhances the splicing efficiency and stability of genes related to fat browning. The expression of The RNA-binding protein Quaking (QKI) promotes cyclization of genes related to browning and maintain their stability (**a**). Low Adenosine deaminase acting on RNA 1 (ADAR1) content can promote reverse complementary sequences (RCS) complementation and stabilize RNA (**b**). Mannose-Binding Lectin (Mbl) proteins bind to introns around circularized exon (**c**). Created with BioGDP.com.

As a class of closed circular RNAs, circRNAs’ biogenesis and circularization mechanisms are central to understanding their structural stability and functional specificity. The classic loop formation process often requires the regulation of Cis-elements - the pairing of reverse complementary sequences (RCS) in the flanks of cyclized exons to form RNA double strands, which enhances the efficiency of back-splicing (e.g. Alu elements)[96, 97]. Through the study of C. elegans samples, it was suggested that during the cyclization of circRNAs, the reverse complementary sequences (RCS) in the introns drive them to form specific secondary hairpin structures and promote cyclization, which expose or hide some miRNA or protein binding sites[98]. Therefore, in the regulatory process related to fat browning, circRNAs can maintain their structure and functional specificity through RCS. To a certain extent, it may provide support for the stabilization after fat browning. Furthermore, a recent study has shown that when the production of circRNAs of organisms is insufficient, adding RCS of the pre-mRNA can be a good way to increase cyclization efficiency, providing new ideas for the treatment of some diseases[99]. However, the proteins Mbl, QKI, and Adenosine deaminase 1 acting on RNA (ADAR1) have been shown to influence the complementarity of RNA pairs in this structure[85, 86, 100, 101]. Above all, controlling the function of these RBP may maintain the stability of metabolism-related circRNAs function to a certain extent. A summary of the findings is presented in Fig. 5. Although the circularization of circRNAs has been of great concern for some years, there is still no more precise artificial circularization procedure available to produce them. Some of them still require further clinical trials to ensure the safety and long-term efficacy of the RNAs produced through this process as drugs. This is a process that requires a long-term accumulation of knowledge and experience.

### CircRNAs affect fat browning by regulating organelle function

Mitochondria are closely related to the energy metabolism and heat production of fat browning[102]. These processes involve fatty acid oxidation, mitochondrial biosynthesis, mitochondrial quality control, mitochondrial morphological changes, mitophagy[103], etc. A recent study indicated that changes in mitochondrial morphology are associated with the activation state of BAT. In mice who lack the MCJ mitochondrial transmembrane protein, the mitochondria in brown adipocytes tend to be rounder, with their function strengthened[104]. Mitophagy is the process of clearing dysfunctional and suboptimal mitochondria, which can be a way to promote the browning of white adipocytes[105, 106]. During the fat browning process, suitable mitophagy is beneficial for maintaining energy metabolism and thermogenesis. In a study by Mottillo et al.[107] specific knockout of the AMPK gene in adipocytes which is critical to maintaining mitochondrial integrity and promoting mitophagy leads to cold intolerance and resistance to β-adrenergic stimulation, thus preserving the quality and function of BAT. Feng et al.[108] induced mitophagy toxicity by constructing a corresponding circular RNA in vitro. Furthermore, some circRNAs influence mitophagy by encoding specific peptides. In a research about functions of mitochondria, cIGF1R (circular RNA IGF1R) encodes C-IGF1R peptides, which interact with VDAC1 to inhibit its ubiquitination, reducing mitophagy[109]. Besides, other studies have also indicated that circRNAs can act as competitive endogenous RNAs. Based on the study by Xie et al.[110] circERCC2 binds to miR-182-5p, which prevents miR-182-5p from inhibiting the expression of its target gene SIRT1, finally inhibiting apoptosis of NCPs and promoting mitophagy.

Not only do mitochondria play the role of thermogenesis and energy consumption in fat browning, but endoplasmic reticulum (ER) stress also plays a significant role in fat metabolism[111]. A study has shown under ER stress, the IRE1α downregulates the expression of the PGC1α through its dependent mRNA degradation (RIDD) mechanism, thereby inhibiting the conversion and oxidation capacity of adipocytes[112]. Besides, A novel study pointed out that ER stress might lead to the activation of the PEPK signaling pathway, leading to the phosphorylation of eIF2α, thereby affecting thermogenesis in BAT[104]. Besides, circHIPK2 in human lung fibroblasts (HPF-a) is involved in regulating σ-1R, which results in ER stress affecting the eIF2α pathway[113]. The latest research indicates that the phosphorylated form of eIF2α (p-eIF2α) can migrate to the outer mitochondrial membrane during stress and directly regulate mitochondrial autophagy[114]. Relevant studies revealed that lipid overloading-induced ER stress not only inhibits the expression of PGC-1α and circRNA SCAR through CHOP, but also leads to mitochondrial dysfunction and elevated mROS levels[115], thus further aggravating ER stress. The interaction of mitochondria and endoplasmic reticulum regulates the homeostasis of energy metabolism, which is the fulcrum of fat browning (Fig. 2). However, it is worth noting that currently, there is still a lack of sufficient information regarding the role of circRNAs in interacting with organelles to regulate lipid metabolism. Moreover, the specific cellular pathways and sites involved are not yet clear. In the future, more precise exploration and studies on a wider range of organelles will be needed.

## Potential therapeutic implications of circrnas for regulating fat browning for clinical diseases

### Application of circRNAs in metabolic diseases

Disorder of fat metabolism leads to various metabolic syndromes, and circRNAs serve as central regulators in metabolic disease prevention by orchestrating the browning of white adipocytes into beige/brown adipocytes[116]. Their molecular functions primarily involve competitive miRNA binding, modulation of protein activity, or translation of functional peptides, thereby activating key browning pathways such as PGC-1α, UCP1, and PRDM16. During obesity development, circTshz2-1 and circArhgap5-2 is important factors for fat synthesis[79]. Concurrently, circSAMD4A mechanistically binds to miR-138-5p, thereby derepressing EZH2 expression to suppress pathological adipogenesis in obese individuals[67]. Meanwhile, the overexpression of circGlis3 increased the mRNA amount of the insulin gene in both vivo and vitro experiments, promoting insulin secretion by upregulating NeuroD1 and Creb1 through its sponge effect on miR-124-3p, and inhibiting the apoptosis of obesity-induced cells[117]. Additionally, ciRS-7, known to adsorb miR-7, can promote β-cell proliferation and insulin secretion[118], and CircHIPK3, which adsorbs specific miRNAs (such as miR-124-3p and miR-338-3p), regulates β-cell genes. All facts above demonstrate that the sponge effect of circRNAs to enhance insulin sensitivity can be applied in the treatment of type 2 diabetes[119]. The liver plays a crucial role in the digestion, absorption, decomposition, synthesis and transportation of lipids. Disorders of lipid metabolism in the liver can lead to the accumulation of reactive oxygen species (ROS), affecting the function of organelles and thereby inducing non-alcoholic fatty liver disease (NAFLD)[120]. Chen et al.[121] discovered that in humans, circ_0057558 could interact with miR-206 to release the inhibition of the ROCK1/AMPK signaling pathway, thereby relieving NAFLD. In addition, according to Zhao’s study[115], circRNA SCAR whose expression is decreased in NAFLD and NASH was found to be related to metabolic inflammation. In addition to the aforementioned metabolic diseases related to the human body, current research is also focusing on the therapeutic significance of circRNAs for ketosis in dairy cows. They found that circ-30628 and circ-CoQ2 are key regulatory molecules in the muscle tissues of ketosis dairy cows, and they participate in energy metabolism, oxidative stress and cell function regulation through the ceRNA mechanism[122]. Based on this, it is possible to develop early diagnostic markers for ketosis in dairy cows.

Critically, circRNA-driven fat browning exerts systemic effects on ameliorating metabolic disorders. This multifaceted impact primarily manifests in the restoration of energy balance through elevated basal metabolic rates and reduced ectopic lipid deposition, alongside improvements in glucolipid homeostasis, which alleviates metabolic stress within the liver. Furthermore, circRNAs contribute to immunomodulation within adipose tissue microenvironments. For instance, recent research indicates that circNrxn2 can promote M2 macrophage polarization, leading to the secretion of anti-inflammatory cytokines (such as IL-4 and IL-10), as well as factors that promote angiogenesis[67]. This immunomodulatory effect is mediated through activation of the PPARγ signaling pathway. Collectively, this multi-organ synergy underscores the potential of circRNAs as promising therapeutic targets for metabolic syndrome. However, it is worth noting that the understanding of the mechanism by which circRNAs affect metabolic-related diseases is insufficient. In particular, there is a lack of systematic understanding of the upstream and downstream pathways and cross-dialogues of the target gene. This may lead to compensatory effects from unknown molecules during clinical application, which requires further research in the future. Furthermore, during its use as a therapeutic drug, the targeting and off-target effects are also major issues, which are related to the safety and long-term efficacy of circRNAs in clinical applications.

### Application of circRNAs in non-metabolic diseases

The systemic metabolic remodeling and immunomodulatory effects caused by fat browning make it potentially associated with cancer, immune diseases and neurodegenerative diseases. In the regulation of the tumor microenvironment, browning adipose tissue limits the energy supply of solid tumors such as breast cancer and colorectal cancer by competitively consuming glucose and free fatty acids[123]. Fei et al.[124] discovered that circ-ITCH can act as a cell cycle inhibitor in esophageal squamous cell carcinoma to suppress tumor growth. CircRNAs PDE8A promotes pancreatic cancer invasion through the miR-338/MACC1/MET signaling pathway[125]. Additionally, circRNAs are involved in regulating the medical resistance of cancer cells through autophagy[126]. It is worth noting that in tumor treatment, circRNAs are often regarded as novel targets or biomarkers. Furthermore, there is a special type of liver cancer (HCC) that is closely related to fat metabolism disorders in animals. From an epidemiological perspective, metabolic dysfunction-associated steatotic liver disease (MASLD) induced by obesity, diabetes, insulin resistance, etc. is on the rise [127, 128]. The abnormal fat metabolism itself can drive the occurrence of this liver cancer[129, 130]. During the process of NAFLD turning to hepatocellular carcinoma (HCC)[129], downregulation of circRNA FTX modulates the polarization of M1/2[130]. Therefore, FTX could be used as a therapeutic target to reduce HCC risk. Apart from this function, circRNAs are being applied as a novel biomarker in precise medicine of various diseases due to their structural stability and disease relevance. Hu et al.[131] found that circPHLPP2 is upregulated in colorectal cancer patients resistant to PD-1 therapy, which confirms it can serve as a prognostic biomarker. They can be utilized in early diagnosis, prognosis assessment, effect monitoring, and other stages[132–134]. At present, there are also numerous studies focusing on the therapeutic significance of circRNAs for different types of tumors.

In addition, brown adipocytes have an inherent advantage for autoimmune diseases. Adipose tissue, as a common protective tissue, plays a significant role in inflammation repair and other aspects. Recent studies have shown that in normal BAT transplanted mice, pro-inflammatory cytokines IL-12, IL-17, IL-6 and tumor necrosis factor-α (TNF-α) tend to decrease, and the level of the PI3K-AKT signaling pathway is lower compared with that in BAT transplanted mice with rheumatoid arthritis (RA)[135]. In addition, BAT secretes adiponectin and IL-6[136], as well as fibroblast growth factor 21 (FGF-21)[137], which can exert anti-inflammatory effects. According to a recent research, circHIPK3 can interact with miR-558 to affect the NF-kB pathway[138], modulating endothelial cell function, to reduce vascular leakage and inflammation and protect blood vessels. In summary, circRNAs induce the fat Browning, promoting the secretion of anti-inflammatory factors by brown adipocytes, altering the inflammatory microenvironment of the body, and having regulatory effects on some immune diseases.

Apart from the treatment of the above-mentioned tumors and immune diseases, circRNAs regulating fat Browning can also indirectly assist in the treatment of neurodegenerative diseases (NDs). In nerve cells, neuroinflammation, mitochondrial dysfunction, and abnormal protein deposition are all pathological features of neurodegenerative diseases[139]. However, promoting fat Browning can facilitate the release of anti-inflammatory factors, maintain the normal functions of mitochondria and proteins, and has indirect therapeutic significance for these diseases. Previous research on Alzheimer’s disease found that through RNA deep sequencing of APP/PS1 mice and wild-type mice, a total of 243 circRNAs transcripts were significantly dysregulated at 9 months[140]. Among them, abnormal interaction between circHomer1 and Homer1b, accompanied by synaptic dysfunction of nerve cells, can be used in the early diagnosis process of neurodegenerative diseases[134]. In amyotrophic lateral sclerosis (ALS), circ-Hdgfrp3 is involved in the regulation of fusion sarcoma protein (FUS)[141], which is related to mitochondrial function and protein accumulation. The key gene UCP-1 for fat Browning is also closely related to the main function of mitochondria, and there may be a certain connection between the two. However, at present, there is still insufficient evidence of the association between circRNAs regulating fat Browning and neurodegenerative diseases. Therefore, further exploration is needed.

## Intervention strategies of circrnas and their potential applications

Currently, the precise application strategies for circRNAs are continuously being refined alongside advancements in science and technology. As a novel type of non-coding RNA, circRNAs possess a unique covalently closed circular structure, which endows them with much higher stability than linear RNAs. Therefore, they can persist for a long time in body fluids and clinical samples. With the development of high-throughput sequencing technology, many circRNAs are believed to be able to exist stably for a long time in serum, exosomes, urine[142], etc. This outstanding stability makes it applicable for clinical diagnosis, especially circRNAs in body fluids and samples, which are novel biomarkers. Exosomal circRNAs have been proposed as biomarkers for cancer early diagnosis and drug development vehicles for diseases[143]. Recent study has shown that exosomal circ_0008285 in the follicular fluid of patients with polycystic ovary syndrome (PCOS) reduces the expression of low-density lipoprotein receptor (LDLR) and influences cholesterol metabolism in ovarian granulosa cells through binding to miR-4644[144]. Besides, through exosomes, the circRNAs-miRNAs-mRNAs axis regulates the role of gut microbes (e.g.circ0000730-miR466i-3p-SOX9 axis)[145]. Specific circRNAs affect nutrient intake and physiological processes of gut microbes, which is a new insight for the treatment of metabolic diseases[146, 147]. For some metabolic diseases, circRNAs have certain application prospects as biomarkers. Recent studies on obese type 2 diabetes have found that through high-throughput sequencing of peripheral blood samples from different humans, 442 circRNAs are differentially expressed in patients compared to healthy individuals[148]. These RNAs often have application prospects as biomarkers. It is also worth noting that circRNAs, as biomarkers of metabolic diseases, have inherent advantages over those of other diseases. Firstly, common metabolic diseases often have a long pathological process. The stability of circRNAs can support the long-term monitoring of metabolic diseases and be used for early diagnosis. By taking blood samples from the early stage of human pregnancy, researchers found that hsa_circRNA_0039480 was highly expressed and had the potential for the early diagnosis of gestational diabetes mellitus (GDM)[149]. Meanwhile, the sampling of cerebrospinal fluid samples for metabolic diseases is more convenient compared to that for neurodegenerative diseases[150], body fluid tests can meet the requirements mostly. Finally, the core pathology of metabolic diseases often occurs in specific organs (adipose tissue, liver, islets), and related circRNAs are often specifically expressed in them. Their specificity is easier to detect compared to cancer markers in multiple organs. Recently, Liu et al.[117] found that circGlis3 is a key factor in obesity-mediated pancreatic islet β-cell dysfunction, and its specificity in the islets makes it have the application prospect as a biomarker. With the advancement of technology, circRNAs have been engineered in vitro to enhance the expression efficiency of proteins associated with them and improve their sustained translation and functional activity within organisms, which is important in trans-organ therapy[95]. However, circRNAs still have certain limitations as biomarkers. As a clinical biomarker, its low abundance is the main obstacle, which relies on highly sensitive techniques for detection and has an impact on the accuracy of quantification[151]. Therefore, more research support for it is needed in the future.

Due to their stability in the bloodstream, circRNAs have potential for use in the development of disease vaccines[152]. CircRNAs vaccines represent a novel vaccine technology characterized by efficient expression and long-lasting immune protection[153]. Research indicates that circRNAs vaccines can be administered directly into the body via delivery systems, utilizing the host cell’s translation machinery to produce antigenic proteins. Long non-coding RNAs (lncRNAs) as a kind of an RNA molecule that is over 200 nucleotides long but does not encode a protein[154], it has important significance in cancer therapy. A study focused on the highly expressed lncRNA H19 in glioblastoma. The scientists in this study developed a vaccine named circH19-vac, which expresses peptides encoded by long non-coding RNA (lncRNA) as tumor antigens to inhibit tumor growth[155]. Therefore, in certain metabolic-related diseases, particularly tumors, specific circRNAs can be experimentally employed as preventive vaccines. Apart from being used as vaccines, synthetic circular interfering RNAs (siRNAs) are an important type in current small nucleic acid therapy. They have overcome the drawback of traditional siRNAs being easily degraded by exonucleases[152]. Researchers have discovered that by combining single-stranded circRNAs with their complementary linear antisense RNA strands, it is beneficial to overcome the off-target effect of siRNAs. This finding has long-term research significance for the long-term efficacy of siRNA-based treatments in the future[156]. The latest clinical data also indicate the promising application prospects of siRNAs in the field of obesity treatment. Liang et al.[157] used siRNA technology to target the key intestinal enzyme SOAT2 involved in lipid absorption, thereby intervening in the development of obesity.

Delivery systems are one of the key technologies for circRNAs as drugs. Commonly used delivery systems include lipid nanoparticles (LNPs), viral vectors, and exosomes[158]. LNPs are characterized by their efficient delivery and low immunogenicity, relatively, making them widely applicable in the development of circRNAs vaccines and therapeutic agents[159]. Studies have shown that multi-armed ionizable lipids and AX4-based lipid nanoparticles (AX4-LNP) demonstrate effective delivery in circRNAs vaccines[160]. However, LNPs delivery system still has significant limitations. Firstly, its ionizable cationic lipids may activate the innate immune response, so repeated high-dose administration can cause inflammation[161]. Moreover, some organs have insufficient targeting, especially non-liver organs, which may lead to off-target effects[162]. Additionally, exosomes, as a natural delivery system, offer excellent biocompatibility and targeting capabilities for circRNAs delivery[163]. However, the difficulty in purifying exosomes and the drug loading capacity remain a major problem that troubles scientists. Consequently, circRNAs have been confirmed as potential drug carriers, facilitating drug delivery and release. This is attributed to their stability and biocompatibility, allowing them to bind with drug molecules for targeted delivery. Furthermore, circRNAs may also be employed in protein replacement therapies, such as expressing specific proteins to treat rare diseases[164–167]. In addition to being used directly as a drug, circRNAs can also indirectly affect the effects of other drugs, either promoting or inhibiting drug resistance. In the treatment of leukemia, circPAN3 enhances the resistance of leukemia cells to doxorubicin through the miR-153-5p/miR-183-5p/XIAP axis[168]. Additionally, circANKRD28 inhibits the resistance of non-small cell lung cancer (NSCLC) to cisplatin through the miR-221-3p/SOCS3 axis[169]. What is exciting is that both the use of circRNAs as drugs and their functional regulation of other drugs provide new ideas for the future deep clinical application of circRNAs.

**Fig. 6:**
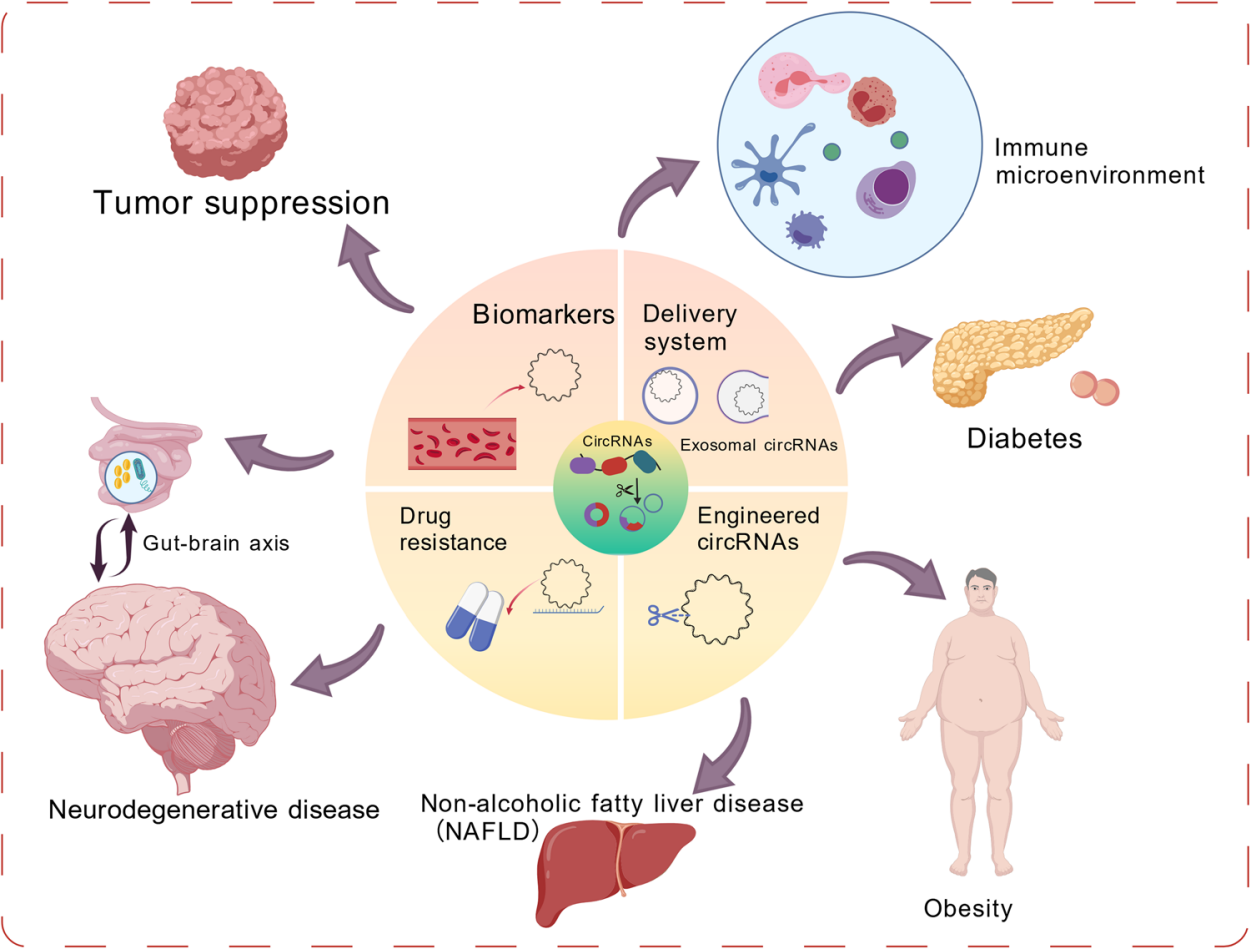
The function of circRNAs, as well as their role in disease and their association with disease treatment. Created with BioGDP.com.

In the field of gene therapy, gene editing technologies like the CRISPR-Cas system can be utilized for circRNAs intervention[170, 171]. By designing specific guide RNAs (gRNAs), the CRISPR-Cas system can precisely cleave circRNAs to regulate their expression. The CRISPR-Cas12a system developed by researchers can be applied for the cleavage and detection of circRNAs[170]. Moreover, gene editing technologies can also facilitate the modification and regulation of circRNAs, such as altering circRNAs sequences through RNA editing techniques[48]. A research team led by Prashant Mali effectively inhibited the expression of the cardiovascular disease-related gene PCSK9 using circRNA-encoded DNA methyltransferase DNMT3A-3L and ZF-KRAB fusion proteins[172]. The application of circRNAs in disease treatment is shown in Fig. 6.

Notably, with the ongoing updates to current databases, some studies have begun to leverage artificial intelligence to predict the functions of circRNAs, employing matrix factorization (MF) and model construction to compute these functions, which is crucial in early disease research[173, 174]. Moving forward, we eagerly anticipate the intervention strategies and potential applications of circRNAs in metabolic diseases within the context of artificial intelligence[175].

## Limitations of the application of circRNAs

Although the closed structure of circRNAs gives them an inherent advantage in resisting external conditions, they still face a bottleneck of timeliness in actual clinical applications. During acute events of myocardial infarction, circRNAs show a rapid and transient surge, and this transient expression limits their application as long-term markers[176]. At the same time, most current studies are only at the stage of exploring basic mechanisms, confirming the regulatory role of knocking out or overexpressing certain specific circRNAs in the life activities of fat browning, but their long-term effects on cells and animals have not been evaluated, which will be the direction of future research.

Although the regulatory role of non-coding RNAs in adipose tissue and adipose browning has been systematically reviewed[177, 178], the elaboration of circRNAs is less than that of other types, and there is insufficient mechanism depth and relatively insufficient clinical conversion prospects. The biggest problem in clinical conversion is the cross-species conservation of circRNAs. It is worth noting that current research on circRNAs is mainly based on experimental mice or other animals, and there is insufficient research on real human samples. This significant difference in conservation between species greatly restricts the universality of mechanism research. Analysis by the CircAge database shows that only about 23% of circRNAs are conserved among seven species[179], while most fat browning-related circRNAs show species-specific expression. Recent studies have found that circSAMD4A in high-fat obese mice can promote fat browning and improve obesity symptoms, and the related human homologous gene mmu_circ_0000529 has been identified[67], which has a high sequence match with this RNA, but whether it has a similar function to the circSAMD4A in mice is still unclear. In the future, the establishment of humanized adipose tissue models (such as organoids) may be able to make up for species differences to a certain extent. In conclusion, although currently there are many studies on the connection between fat browning and circRNAs, there is a lack of clinical relevance in human evidence, or only human cell samples are used for in vitro experiments or deep sequencing, and the number of sample repetitions is insufficient. Future research needs to focus on these issues.

## Conclusions and prospects

In summary, promoting the browning of adipocytes became a new direction in the treatment of fat-related diseases, with the potential of circRNAs gradually emerging as a novel class of non-coding RNAs, circRNAs regulate the process of fat metabolism through their sponge effect, interaction with proteins, and encoding functions. They demonstrate significant potential, especially in promoting fat browning, enhancing energy expenditure, and regulating organelle function. However, some of their more detailed mechanisms remain to be clarified. Currently, based on the functions of circRNAs, researchers have been able to modulate relevant biological activities through gene knockout and engineered modification of them.

Future studies should further explore how circRNAs influence fat metabolism and browning processes through interactions with key genes, transcription factors, and signaling pathways. Furthermore, attention should be paid to the improvement of its safety as a biomarker, the avoidance of off-target effects, and the enhancement of its long-term efficacy during the drug delivery process. The cross-species gene conservation aspect is also a major focus. In the future, the combination of organoid development may provide more human evidence for the development of this field.

**Fig. 7:**
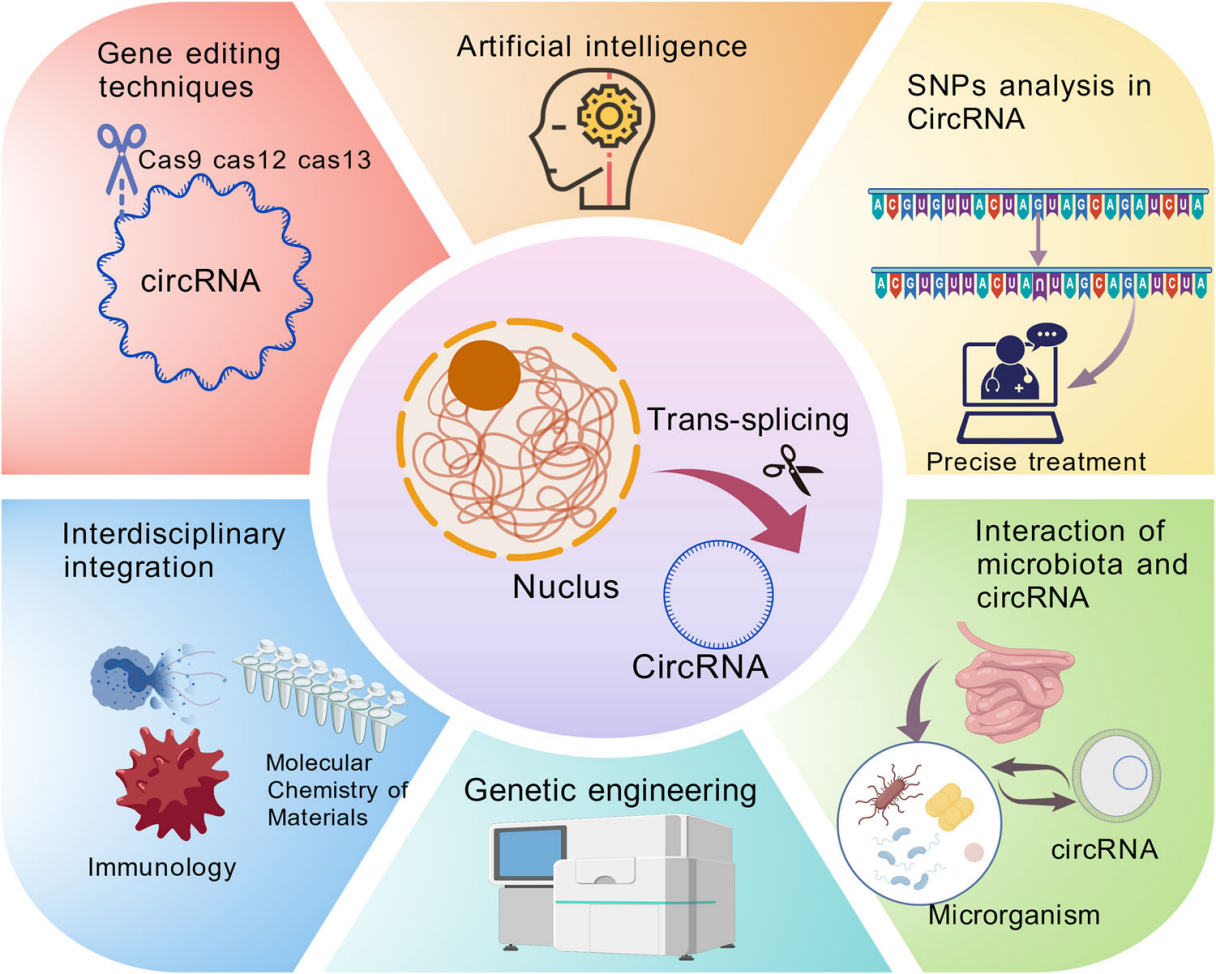
Key development directions and technical prospects of circRNAs. Prospects for the development of circRNAs, including genetic engineering, gene editing, application of AI, detection of SNPs, and interaction between microorganisms and circRNAs. Created with BioGDP.com.

By integrating multi-omics technologies, new biomarkers and therapeutic targets may be identified, offering precision treatment strategies for metabolic diseases such as obesity, type 2 diabetes, and NAFLD. Besides, in the era of molecular biology, more novel techniques will be applied in the research of circRNAs in the future. The translational potential of circRNAs regulatory networks in the treatment of metabolic diseases: use gene editing techniques (such as CRISPR/Cas9, CRISPR/Cas12, CRISPR/Cas13, and antisense nucleotide) to specifically modify, knock out, or overexpress RNA molecular fragments to Obesity or insulin resistance[170, 171, 180]. Using artificial intelligence to develop a deep learning-based circRNAs functional prediction model: combining matrix factorization and neural networks (MFNN) to efficiently predict the potential effects of unknown RNA-RBP on fat metabolism[173]. AI-assisted analysis between SNPs and circRNAs can facilitate the identification and removal of pathogenic variants, thereby enabling personalized disease treatment approaches[181]. The metabolic typing strategy of circRNAs expression profile in single cells is utilized for individualized treatment of patients with metabolic diseases[182]. The interaction between circRNAs and gastrointestinal-associated flora may be regulated by a targeted delivery system of special materials[147, 183]. Novel delivery systems of circRNAs (e,g, LNPs or exosomes) and drug development[152]. Establish a platform for multidisciplinary integration (e.g., immunology, materials science) to conduct research on the optimization of vaccines and disease surveillance. The future development prospects of circRNAs application are shown in Fig. 7.

In conclusion, it is hoped that with continuous advancements in technology and deepening exploration, a more comprehensive understanding of the mechanisms underlying fat browning will emerge, and these theoretical insights will be transferred into practical clinical applications, providing new approaches for the treatment of fat-related diseases in the future.

## Data Availability

Data sharing is not applicable to this article as no datasets were generated or analyzed during the current study. No new data and scripts were generated for this review.
